# Fetal exome sequencing for isolated increased nuchal translucency: should we be doing it?

**DOI:** 10.1111/1471-0528.16869

**Published:** 2021-09-14

**Authors:** R Mellis, RY Eberhardt, SJ Hamilton, The PAGE Consortium, DJ McMullan, MD Kilby, ER Maher, ME Hurles, JL Giordano, V Aggarwal, DB Goldstein, RJ Wapner, LS Chitty

**Affiliations:** ^1^ Genetics and Genomic Medicine UCL Great Ormond Street Institute of Child Health London UK; ^2^ NHS North Thames Genomic Laboratory Hub, Great Ormond Street Hospital for Children NHS Foundation Trust London UK; ^3^ Wellcome Sanger Institute Hinxton UK; ^4^ NHS Central and South Genomic Laboratory Hub Birmingham Women's and Children's NHS Foundation Trust Birmingham UK; ^5^ Fetal Medicine Centre Birmingham Women's and Children's NHS Foundation Trust Birmingham UK; ^6^ Institute of Metabolism and Systems Research, College of Medical Sciences University of Birmingham Birmingham UK; ^7^ Department of Medical Genetics University of Cambridge Cambridge UK; ^8^ Department of Clinical Genetics Cambridge University Hospitals NHS Foundation Trust Cambridge UK; ^9^ Department of OBGYN Columbia University Irving Medical Center New York NY USA; ^10^ Department of Pathology and Cell Biology Vagelos College of Physicians and Surgeons New York NY USA; ^11^ Institute for Genomic Medicine Vagelos College of Physicians and Surgeons New York NY USA

**Keywords:** Fetal diagnosis and therapy, Genetics, Perinatal diagnosis‐invasive, Perinatal diagnosis‐ultrasound

## Abstract

**Objective:**

To evaluate the utility of prenatal exome sequencing (ES) for isolated increased nuchal translucency (NT) and to investigate factors that increase diagnostic yield.

**Design:**

Retrospective analysis of data from two prospective cohort studies.

**Setting:**

Fetal medicine centres in the UK and USA.

**Population:**

Fetuses with increased NT ≥3.5 mm at 11–14 weeks of gestation recruited to the Prenatal Assessment of Genomes and Exomes (PAGE) and Columbia fetal whole exome sequencing studies (*n* = 213).

**Methods:**

We grouped cases based on (1) the presence of additional structural abnormalities at presentation in the first trimester or later in pregnancy, and (2) NT measurement at presentation. We compared diagnostic rates between groups using Fisher exact test.

**Main outcome measures:**

Detection of diagnostic genetic variants considered to have caused the observed fetal structural anomaly.

**Results:**

Diagnostic variants were detected in 12 (22.2%) of 54 fetuses presenting with non‐isolated increased NT, 12 (32.4%) of 37 fetuses with isolated increased NT in the first trimester and additional abnormalities later in pregnancy, and 2 (1.8%) of 111 fetuses with isolated increased NT in the first trimester and no other abnormalities on subsequent scans. Diagnostic rate also increased with increasing size of NT.

**Conclusions:**

The diagnostic yield of prenatal ES is low for fetuses with isolated increased NT but significantly higher where there are additional structural anomalies. Prenatal ES may not be appropriate for truly isolated increased NT but timely, careful ultrasound scanning to identify other anomalies emerging later can direct testing to focus where there is a higher likelihood of diagnosis.

## Tweetable abstract

Prenatal ES has a low diagnostic rate (<2%) for isolated increased NT but is significantly more likely to yield a diagnosis where there are additional fetal structural anomalies.

**Linked article** This article is commented on by AN Talati and NL Vora, p. 61–62 in this issue. To view this mini commentary visit https://doi.org/10.1111/1471-0528.16942.

## Introduction

An increased nuchal translucency (NT) >3.5 mm detected at first‐trimester ultrasound screening is associated with fetal chromosomal abnormalities, structural anomalies (such as congenital heart malformations), and a wide range of genetic disorders.^1^, ^2^ Investigation of fetuses with increased NT typically comprises rapid aneuploidy testing and chromosomal microarray (CMA) on a fetal DNA sample obtained through chorionic villus sampling or amniocentesis. A chromosomal abnormality will be identified in approximately 30% of cases^3^, ^4^ but euploid fetuses with increased NT remain at increased risk of adverse outcomes, proportionally related to the degree of NT enlargement.^2^, ^3^, ^4^

In chromosomally normal fetuses with structural anomalies, prenatal exome sequencing (ES) has been shown to increase the diagnosis of monogenic conditions, with diagnostic rates varying widely across different phenotypes.^5^, ^6^, ^7^, ^8^ Two large, prospective studies of unselected fetuses with any structural abnormality showed that ES provided additional diagnosis in 8.5% and 10.3% of cases, respectively.^5^, ^6^ However, in fetuses with multisystem or skeletal abnormalities the diagnostic rate was over 15% whereas in fetuses with isolated increased NT (≥3.5 mm) the diagnostic rates were only 3.2% and 2.9%, respectively.^5^, ^6^ Similar low diagnostic rates have also been reported recently for isolated increased NT,^9^, ^10^ bringing into question the clinical utility or cost‐effectiveness of prenatal ES in this situation.

With increasing availability of sequencing technology, decreasing costs and improved speed of bioinformatic analytical pipelines, rapid fetal ES for prenatal diagnosis is moving beyond the research arena and has recently been implemented in the UK National Health Service (NHS) and in many prenatal diagnosis centres across the USA and Europe. A clear evidence‐base is required to enable the most efficient use of this new technology. Here we review the final, extended data sets of the UK Prenatal Assessment of Genomes and Exomes (PAGE) and USA Columbia (CUIMC) studies to identify all cases presenting with increased NT, aiming to further delineate which pregnancies benefit most from prenatal ES. We review natural histories, outcomes and diagnostic variants and explore factors influencing diagnostic yields to inform further development of guidelines for the use of prenatal ES in the presence of increased NT in clinical practice.

## Methods

The study cohort comprised fetuses presenting with increased NT (≥3.5 mm) recruited to the PAGE^5^ and CUIMC fetal whole exome sequencing^6^ studies. The PAGE study defined increased NT as ≥4.0 mm on first‐trimester ultrasound scanning, measured according to UK NHS Fetal Anomaly Screening Programme criteria.^11^ The CUIMC study defined increased NT as ≥3.5 mm, measured according to Nuchal Translucency Quality Review criteria.^12^ In the PAGE study there was consecutive recruitment of the first 100 cases presenting with isolated increased NT, as recruitment of cases in any specific category was capped at 10% of the total target cohort of 1000 or ˜20% of the running total.

### PAGE study

Here we review 876 fetuses and 1727 matched parental samples (851 fetus–parent trios and 25 fetus–parent duos), of which 610 cases (596 trios and 14 duos) have been reported.^5^ Study methodology and eligibility criteria were as previously published^5^ but in brief, couples undergoing invasive testing for any ultrasound identified fetal abnormality, including isolated increased NT, were consented for trio ES where fetal karyotype/CMA were normal. Whole exome sequencing was performed with analysis targeted to a virtual panel of 1628 genes associated with developmental disorders.

### CUIMC study

CUIMC recruited a total of 494 fetuses with matched parental samples, of which 234 trios have been reported.^6^ The study consented parents with pregnancies complicated by any fetal abnormality, including isolated increased NT, for invasive testing or collection of a cord sample after birth. Untargeted trio whole exome sequencing was performed when karyotype/CMA was non‐causative of the anomaly. The bioinformatic analysis is described elsewhere.^6^

### Variant interpretation

In both studies, candidate pathogenic variants were curated and discussed in consensus with relevant clinicians and scientists at a multidisciplinary clinical review panel (CRP). Only variants classified as ‘pathogenic’ or ‘likely pathogenic’ according to American College of Medical Genetics and Genomics guidelines^13^ and judged likely to cause the observed structurally abnormal phenotype in the fetus were considered as positive diagnostic results, validated using Sanger sequencing and reported to parents after delivery in the PAGE study or at the time of diagnosis in the CUIMC study.^5^, ^6^

### Procedures

Interrogation of the study databases identified all fetal cases presenting at 11–14 weeks of gestation with any of the following terms recorded: ‘Increased nuchal translucency’ (HP:0010880); ‘Fetal cystic hygroma’ (HP:0010878); ‘Cystic hygroma’ (HP:0000476); ‘Thickened nuchal skin fold’ (HP:0000474), whether in isolation or in combination with other phenotypes. For the purpose of this analysis, no distinction was made between increased NT and cystic hygroma, which can also be described as a septated increased nuchal translucency, on the basis that practitioners documenting the fetal phenotype at the time of recruitment may have used the terms interchangeably and we sought to capture all relevant cases from the study databases.

To ascertain cases with isolated increased NT at presentation, clinical information was manually reviewed, including the phenotypes (Human Phenotype Ontology terms and free text) recorded in the study databases and ultrasound scan reports at presentation. Following manual review of this information, any fetus without other structural anomalies at the point of presentation (including the absence of so‐called ‘soft markers’, such as short femurs or absent/hypoplastic nasal bone) was classified as ‘initially isolated increased NT’. Of note, both cohorts included some cases previously classified and published in other phenotypic groups as those classifications were originally based upon the predominant phenotype in the pregnancy as a whole, whereas here the classifications are based specifically upon the phenotype at initial presentation at 11–14 weeks of gestation.

For all cases with initially isolated increased NT at presentation, further ultrasound scan reports and clinical information from later in pregnancy were reviewed to ascertain whether the increased NT resolved, remained isolated, or if additional structural abnormalities were detected at a later gestation. Cases presenting with features consistent with established or evolving fetal hydrops (generalised oedema, pleural or pericardial effusions, ascites) were classed as non‐isolated increased NT, as fetal hydrops is a distinct clinical entity with different prognostic implications from isolated increased NT. Pregnancy outcomes, and postnatal clinical information or post‐mortem findings were ascertained from participating fetal medicine units.

### Outcomes

The primary outcome assessed in both this and the previously published studies^5^, ^6^ was the detection of diagnostic genetic variants considered to have caused the observed fetal structural anomaly. We reviewed the exome sequence variants identified in the PAGE and CUIMC studies in this increased NT cohort and calculated diagnostic rates for fetuses with: (1) non‐isolated increased NT at presentation; (2) initially isolated increased NT with additional abnormalities detected later in pregnancy; and (3) isolated increased NT that remained isolated or resolved. We also calculated diagnostic rates according to the measured thickness of NT at presentation.

### Statistical analysis

The two‐tailed Fisher’s exact test was used to compare rates of diagnostic genetic variants between sub‐groups and Bonferroni correction for multiple testing was applied.

### Patient involvement

Design and conduct of the PAGE study was informed by input from patients and the public through collaboration with the charity Antenatal Results and Choices. The CUIMC study was designed and implemented by faculties of the Department of OBGYN and the Institute for Genomic Medicine. There was no additional patient involvement for the analysis presented here.

## Results

In total, 213 fetuses with increased NT at 11–14 weeks of gestation were identified; 159 were classified as initially isolated, whereas 54 had additional structural abnormalities or fetal hydrops at presentation (in the first trimester). Following review and classification of candidate variants by the multidisciplinary CRPs of the studies, 28 (13.1%) of 213 cases had a diagnostic variant identified (Tables 1 and 2).

**Table 1:** Diagnostic variants identified after trio ES and review by PAGE/CUIMC study CRP in fetuses initially presenting with non‐isolated increased NT at 11–14 weeks of gestation

Study ID	NT (mm)	Additional findings at presentation	Findings at later scans	Variant(s) [Inheritance]	ACMG class
PP0342	8.0	Hydrops; dysmorphic facies; arthrogryposis	N/A (ToP)	*CHRNG* c.1010_1011del p.(His337Leufs*60) [Mat]** *CHRNG* c.459dup p.(Val154Serfs*24) [Pat]**	P P
PP3174	4.1	Rhizomelia	N/A (IUD)	*TRIP11* c.757C>T p.(Arg253*) [Hom]	LP
PP1780	8.6	Encephalocele; hypoplastic thorax; TR; polycystic dysplastic kidneys; polydactyly; bilateral talipes; short long bones	As at presentation	*TCTN2* c.1506‐2A>G [Hom]**	P
PP2567	9.7	Mild ascites	CCAM right lung; hydrops	*PTPN11* c.922A>G p.(Asn308Asp) [Mat]** *SOS1* pathogenic variant [Pat] additionally detected on a postnatal RASopathy panel	P
PP2000	9.3	Bilateral talipes; clenched hands	N/A (IUD)	*RYR1* c.7826C>A p.(Ser2609*) [Mat]** *RYR1* c.10177_10198del p.(Leu3393CysfsTer25) [Pat]**	LP
PP4147	5.0	Cystic hygroma; oedema; polydactyly; bright kidneys; encephalocele	N/A (ToP)	*TCTN3* c.628‐13_643del (splice variant) [Hom]	P
PP3324	6.1	Septated cystic hygroma; hydrops	N/A (ToP)	*BRAF* c.1782T>G p.(Asp594Glu) [De novo Het]	LP
PP1843	11.3	Hydrops	Horseshoe kidney; Borderline VM; mid‐face hypoplasia	*KMT2D* c.6295C>T p.(Arg2099*) [De novo Het]**	P
PP3732	19.0	Cystic hygroma; fixed flexed extremities; no stomach or bladder seen	N/A (ToP)	*RYR1* 420bp deletion encompassing exon 29 [Hom]	LP
PP3393	5.1	Overriding aorta (suspected tetralogy of Fallot)	Pericardial effusion; VSD	*GPC3* c.677delC p.(Thr226Ilefs*8) [Mat Hemi]	LP
PP4393	N/S	Cystic hygroma; hydrops; fixed flexed extremities; fetal akinesia sequence	N/A (ToP)	*RYR1* c.8342_8343delTA p.(Ile2781Argfs*49) [Pat] *RYR1* c.2045G>A p.(Arg682Gln) [Mat]	P
Fetal0183	3.5	Micromelia; micrognathia; talipes; ambiguous genitalia	N/A (ToP)	*COL2A1* c.1358G>T p.(Gly453Val) [De novo Het]**	P

ACMG, American College of Medical Genetics and Genomics; CCAM, congenital cystic adenomatous malformation; Hemi, hemizygous; Het, heterozygous; Hom, homozygous; IUD, in utero death; LP, likely pathogenic; Mat, maternal; N/A, not applicable; N/S, not specified; P, pathogenic; Pat, paternal; ToP, termination of pregnancy; TR, tricuspid regurgitation; VM, ventriculomegaly; VSD, ventricular septal defect.

**Variants previously published.

**Table 2:** Diagnostic variants identified after trio ES and review in fetuses presenting with initially isolated increased NT at 11–14 weeks of gestation

Study ID	NT (mm)	Additional findings at presentation	Findings at later scans	Variant(s)	ACMG class
Fetuses with initially isolated increased NT, then other anomalies detected later					
PP2904	9.5	None	Hydrops; ASD	*EPHB4* c.759dupC p.(Ser254Glnfs*10) [De novo Het]**	LP
PP1726	8.0	None	Narrowing of aorta, suspected coarctation	*TAB2 c.1311*_1312delTC p.(Pro438Glnfs*2) [De novo Het]**	LP
PP0503	4.5	None	AVSD	*PTPN11* c.922A>G p.(Asn308Asp) [Mat]**	P
PP0692	6.0	None	Short limbs, polyhydramnios	*RAF1* c.786T>G p.(Asn262Lys) [De novo Het]	LP
PP1864	7.4	None	Hypoplastic left heart syndrome with DORV, TGA, PA	*KMT2D* c.673+1G>A [De novo Het]**	LP
PP2033	6.5	None	Hypoplastic left heart syndrome with DORV	*CHD7* c.656dupG p.(Leu220Profs*67) [De novo Het]**	LP
PP1462	8.9	None	Short femurs; cystic dilatation of lymphatics from neck to upper chest; bilateral RPD	*BRAF* c.770A>G p.(Gln257Arg) [De novo Het]**	P
PP1807	4.7	None	Hypoplastic right heart; VSD	*MID1* c.1102C>T p.(Arg368*) [De novo Hemi]**	P
Fetal0116	N/S	None	Hydrocephalus; hyperflexed feet NB: Couple had previous pregnancies similarly affected	*FLVCR2* c.1509+1G>A (splice variant) [Mat]** *FLVCR2* c.1001dupT p.(Met334Ilefs*37) [Pat]**	LP LP
Fetal0222	5.2	None	Pleural effusion; ascites	*SOS1 c.1132A>G* p.(Thr378Ala) [Pat]**	P
Fetal0307	4.7	None	Shones complex	*NR2F2* c.1091delT p.(Leu364Cysfs*15) [De novo Het]	P
Fetal0385	4.7	None	Short long bones; flattened facies; short nasal bone; ambiguous genitalia	*FGD1* c.2026_2028delGAG p.(Glu676del) [Mat Hemi]	LP
Fetuses with initially isolated increased NT which remained isolated or resolved later in pregnancy					
PP0602	4.8	None	None	Chr15 UPD [Mat]**	N/A
Fetal0045	3.5	None	None	*RERE c.248dupA* p.(Ser84Valfs*4) [De novo Het]**	LP
Fetuses presenting with initially isolated increased NT where later pregnancy follow up was not possible					
PP3321	9.9	None	N/A (ToP)	*PTPN11* c.214G>A p.(Ala72Thr) [De novo Het]	LP
PP2039	6.2	None	N/A (IUD)	*NIPBL* c.1435C>T p.(Arg479*) [De novo Het]**	P

ACMG, American College of Medical Genetics and Genomics; ASD, atrial septal defect; AVSD, atrioventricular septal defect; DORV, double outlet right ventricle; Hemi, hemizygous; Het, heterozygous; IUD, in utero death; LP, likely pathogenic; Mat, maternal; N/A, not applicable; N/S, not specified; P, pathogenic; PA, pulmonary atresia; Pat, paternal; RPD, renal pelvis dilatation; TGA, transposition of great arteries; ToP, termination of pregnancy; UPD, uniparental disomy; VSD, ventricular septal defect.

**Variants previously published.

An additional eight variants (Table S1) were designated as ‘potentially clinically relevant’ by the PAGE study CRP, because either there was insufficient evidence to classify the variant as (likely) pathogenic and/or the prenatal phenotype was not specific enough to be unequivocally attributed to the variant. Six of these were in fetuses with additional abnormalities and two in fetuses with isolated increased NT (Table S1). Variants previously published in the PAGE and CUIMC studies^5^, ^6^ are indicated in Tables 1, 2 and S1.

### Fetuses with increased NT and other anomalies

**Figure 1:**
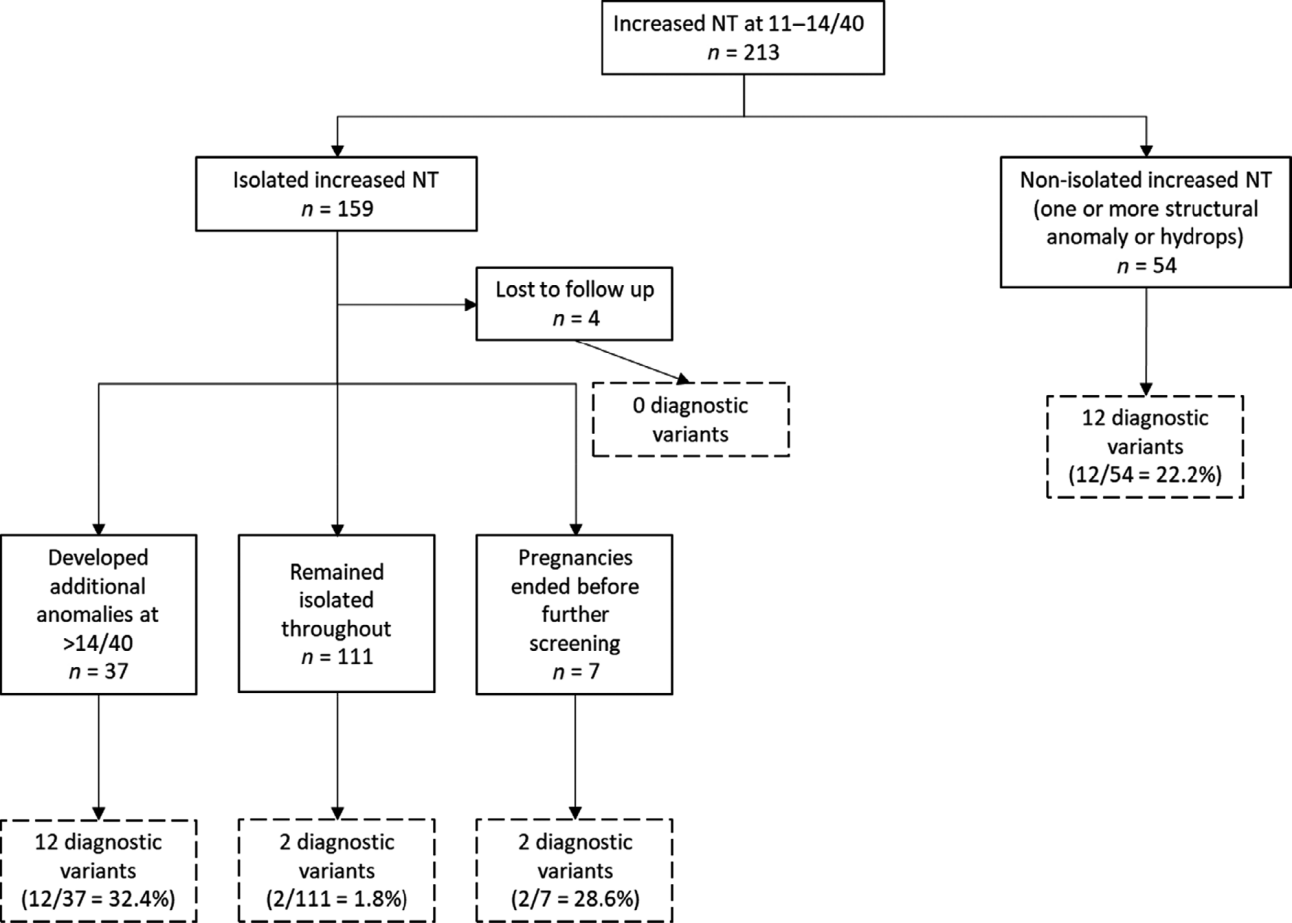
Natural history of pregnancies presenting with increased NT below 14 weeks of gestation.

Diagnostic variants were detected in 12 (22.2%) of 54 fetuses presenting with non‐isolated increased NT (Table 1). Of the 155 pregnancies presenting with initially isolated increased NT and with follow up to term (Fig. 1), additional abnormalities were detected in 37 cases (23.9%) later in pregnancy with diagnostic variants detected in 12 (32.4%). Noonan syndrome accounted for 4/12 (33.3%) of the diagnoses made (Table 2). A further six fetuses had variants designated ‘potentially clinically relevant’, of which 2/6 (33.3%) were also in Noonan syndrome genes (Table S1).

### Fetuses with isolated increased NT

In the 111 cases where no other fetal anomalies developed, and the increased NT either resolved or was not commented on later in pregnancy, a diagnostic variant was detected in two (1.8%) (Table 2). One was a diagnosis of maternal chromosome 15 uniparental disomy, not detected on prenatal CMA, in a fetus presenting with an NT of 4.8 mm at 13 weeks of gestation who was born at term, small for gestational age but with no apparent congenital abnormalities observed on clinical examination (Table S2). The second was a fetus presenting with isolated NT of 3.5 mm and found to have a de novo frameshift variant in the gene *RERE*. This fetus also had no apparent congenital abnormalities at birth, but at 8 months of age had clinical features consistent with *RERE*‐related disease, at which point the prenatally detected variant was reclassified as pathogenic by the study multidisciplinary team. Two further cases had ‘potentially clinically relevant’ variants (Table S1). One, with a variant in *KMT2A,* had a sacral dimple at birth but no other problems were noted on follow up to 2 years of age to allow a diagnosis of Wiedemann–Steiner syndrome to be made. The second had a *KMT2D* variant and although there were no problems detected on clinical examination at birth, examination at 18 months revealed fetal finger pads, arched eyebrows and a sacral dimple, which allowed confirmation of a diagnosis of Kabuki syndrome.

### Fetuses with no follow up

In seven cases, the pregnancies ended soon after the initial presentation with no further scans performed. Diagnostic variants were detected in two (28.6%) of these cases (Table 2). Post‐mortem examination confirmed findings compatible with Cornelia de Lange syndrome in the fetus with a de novo pathogenic *NIPBL* truncating variant. In the other, with a de novo likely pathogenic *PTPN11* variant, post‐mortem confirmed the presence of a cystic hygroma. Four further cases, with no diagnostic variants identified, were lost to follow up and scan reports from later in the pregnancies were not available for review. These 11 cases are excluded from further analysis of diagnostic rates.

Sub‐analysis according to the presence of additional structural abnormalities compared with pregnancies with ‘truly’ isolated increased NT showed a significant increase in the diagnostic rate both where additional abnormalities were seen at presentation (1.8% versus 22.2% *P* < 0.001), and where additional abnormalities developed later (1.8% versus 32.4% *P* < 0.001). There was no statistically significant difference in the frequency of diagnostic variants between fetuses with additional abnormalities at presentation and those developing additional abnormalities at a later gestation (22.2% versus 32.4% *P* = 0.336).

Review of sequencing results in relation to the size of isolated increased NT at presentation (Table 3) showed that diagnostic rate increased with increasing size of NT, from 1.6% (1/63 cases) where NT was between 3.5 and 4.4 mm, to 28.6% (4/14 cases) where NT was >7.5 mm (*P* < 0.05).

**Table 3:** Number of diagnostic variants identified by trio ES in relation to size of isolated NT at presentation

NT (mm)	Number of cases	Diagnostic variants detected (%)
3.5–4.4	63	1 (1.6)
4.5–5.4	42	6 (14.2)
5.5–6.4	22	2 (9.1)
6.5–7.4	11	2 (18.2)
≥7.5	14	4 (28.6)
Not specified	7	1 (14.3)
Total	159	16 (10.1)

## Discussion

### Main findings

In this cohort of pregnancies enrolled in the first trimester with an increased NT of at least 3.5 mm, we observed a relatively low rate of diagnostic variants (1.8%) from prenatal ES for isolated increased NTs that remained isolated throughout the pregnancy. However, there was an increased diagnostic rate where fetuses had additional structural anomalies or hydrops, either at presentation (22.2%) or developing later in pregnancy (32.4%) We also observed significantly higher diagnostic rates where the size of the isolated increased NT was larger at presentation.

It is of note that in the studies we describe there were some likely pathogenic/pathogenic variants that did not explain the fetal phenotype. In line with the study protocols, these variants were not initially reported to the parents. However, postnatal follow up in two cases, a fetus with an *RERE* variant and one with a *KMT2D* variant, revealed an evolving phenotype compatible with these variants and results were reported to parents. These cases highlight one of the limitations of fetal phenotyping, and how with prenatal sequencing we are expanding our understanding of fetal phenotype–genotype relationships previously only recognised postnatally. Documenting this growing knowledge is essential for accurate prenatal interpretation and complete reproductive genetic counselling in future cases.

It is also notable that this cohort includes three diagnoses of Noonan syndrome where causative variants were inherited from undiagnosed affected parents (PP2567, PP0503, fetal0222). In two cases there was a history of previous pregnancy loss with relevant phenotypes (large cystic hygroma and fetal hydrops, respectively), and in two cases the affected parent had unrecognised clinical features of Noonan syndrome. These cases highlight the need for careful review of family and past obstetric history, as well as careful, expert parental examination when considering the underlying aetiology of increased NT to guide molecular testing, particularly where genes exhibit variable penetrance or expression.

### Strengths and limitations

A strength of this study is its relatively large sample size, drawn from the two largest published prenatal ES cohorts to date. Further, the prospectively collected, unselected nature of the cohort, and the detailed approach to examining the natural histories of the pregnancies presenting with isolated increased NT, make this study relevant to clinical practice where rapid ES may be considered in an ongoing pregnancy.

Despite the large size of the PAGE and CUIMC studies, the number of fetuses recruited with (apparently) isolated increased NT is not as high as might be expected because the PAGE study ‘capped’ recruitment of fetuses in any one category at ˜20% of the ongoing total.^5^ Ultimately, it will be beneficial to study much larger cohorts to inform counselling and guide the future use of ES for this group.

Furthermore, varied interpretations of ‘isolated’ increased NT (e.g. isolated at presentation versus isolated throughout the entire pregnancy, and whether or not ‘soft markers’ of genetic abnormality are classed as additional abnormalities) limit comparison of results between studies. A further limitation of prenatal ES for the investigation of isolated increased NT is the difficulty in interpreting genetic variants in the absence of specific fetal phenotypes, exacerbated by a dearth of publically available data regarding the complete spectrum of Mendelian disease in the fetal period.

### Interpretation

Other recent small studies of prenatal trio ES have also observed relatively low diagnostic rates of 0–3% for isolated increased NT,^9^, ^10^ particularly when specifically reporting cases without structural abnormalities developing later in pregnancy.^14^ These low numbers of molecular diagnoses from prenatal ES are consistent with an existing body of evidence indicating that once chromosomal abnormalities are excluded, if detailed follow‐up scanning demonstrates resolution of the increased NT and the absence of any major abnormalities, then the chance of delivering a healthy infant with no major abnormalities is >95%.^1^, ^2^, ^3^ Our observation that diagnoses from prenatal ES increased with enlarging size of NT at presentation is also in keeping with the known association between significant underlying pathogenicity and increasing NT thickness.^1^

In contrast to our findings, a recent smaller retrospectively collected cohort study reported by Choy et al. using prenatal whole genome sequencing reports a diagnostic yield of 17.2% (5/29 cases) among fetuses with isolated increased NT and normal CMA, and found no significant difference between isolated and non‐isolated increased NT groups.^15^ The pathogenic variants reported comprised one case of mosaic Turner syndrome (45,X) not detected on CMA, and four variants in the genes *ARMC4*, *ANKRD11*, *GATA4* and *NSD1*, all of which would have been amenable to detection by whole exome sequencing in the PAGE and CUIMC studies. Differences in the approach to reporting variants may contribute to the difference in diagnostic rates between this and our studies. In the study by Choy et al., findings were not reported back to families, whereas diagnostic findings from the PAGE and CUIMC studies were confirmed in a clinical laboratory and reported to families after the end of the pregnancy.^15^ The CRPs of these studies took a stringent approach to reporting only variants classified (likely) pathogenic and considered causative of the fetal phenotype. With a non‐specific fetal phenotype such as isolated increased NT, it may be challenging to make a definitive genotype–phenotype correlation as well as there being some subjectivity in reporting decisions. This is especially true for novel variants as reported by Choy et al.^15^ This highlights an important point about the need for clear (international) consensus guidelines for reporting variants detected by prenatal ES or whole‐genome sequencing in clinical practice, where results will be largely returned during an ongoing pregnancy and will have implications for counselling and management in that pregnancy.

## Conclusion

These findings have clinical implications for offering prenatal ES in obstetric practice, where testing should aim to maximise benefit to patients without unduly increasing parental anxiety, and are particularly pertinent in view of the recent introduction of rapid fetal exome sequencing in the English NHS.^16^ Trio ES currently remains relatively costly and time consuming and this inevitably plays a role in determining how prenatal ES can be offered. In England, for example, limited numbers of prenatal ES are funded, so cases must fulfil specific eligibility criteria^16^ such that only those with a higher likelihood of a monogenic disorder are tested. Criteria for offering prenatal ES will vary across different healthcare systems but all guidelines must take into account both clinical utility and cost‐effectiveness to direct finite resources appropriately. Until the costs of trio ES fall and testing capacity expands, it is unlikely in a publicly funded healthcare setting that all pregnancies with an increased NT can be offered ES. As diagnostic yield for completely isolated increased NT is low, a suggested strategy is to offer prenatal ES for increased NT only when additional fetal structural abnormalities are present and then offer early detailed scanning to detect emerging anomalies for those with apparently isolated increased NT. Such an approach would integrate well into existing care pathways as many providers already have established protocols for following up isolated increased NT detected at first‐trimester scanning with detailed anomaly scanning and/or fetal echocardiography at 16–18 weeks of gestation. Detecting additional abnormalities before the routine second‐trimester anomaly scan could facilitate completion of ES in a timely fashion. As many of these pregnancies with an increased NT in the first trimester will have undergone chorionic villus sampling for detection of aneuploidy and copy number variations, DNA can be saved at the time of the initial diagnostic testing, which can subsequently be used for ES if second‐trimester ultrasound reveals an emerging phenotype.

In our combined cohort (from two countries), such a strategy would have avoided 116 negative ESs but missed two diagnoses – chromosome 15 uniparental disomy (maternal) in a fetus with isolated increased NT of 4.8 mm at 13 weeks of gestation and normal scans thereafter, and a *RERE*‐related developmental disorder in a fetus with isolated increased NT of 3.5 mm and normal scans thereafter. A further consideration is whether this stepwise strategy would be acceptable to patients, given that detection of increased NT in the first trimester induces anxiety and any wait for further investigations may be stressful. Detailed investigation of this question is beyond the scope of the current study but further research to explore patient perspectives is underway.^17^

As reported by others,^1^ the risk of underlying pathology increases with increasing NT size. In our cohort, 4/14 (28.6%) of cases with an isolated NT ≥7.5 mm in the first trimester had a diagnostic pathogenic variant. The numbers are small and further study is required, but a policy of offering ES for isolated NT of this size may be worth considering.

Where panel testing for RASopathies is available prenatally, this could provide an alternative option for investigating very large isolated increased NT.^18^ The case of Kabuki syndrome described above (PP0722), together with other published evidence,^6^, ^7^, ^15^ demonstrates that Kabuki syndrome can present prenatally with increased NT, so limited analysis for this condition as well as Noonan spectrum disease may be worthy of consideration in the future where significant and persistent isolated increased NT is identified. A potential alternative strategy here to limit costs may be to sequence the fetus alone and investigate parents only where a relevant variant is found in the fetus. Should a limited panel approach be offered, clinicians must provide clear counselling to parents regarding the benefits and limitations of analysing only a small gene set.

Our findings further highlight the significant challenges of variant interpretation in the prenatal setting when the fetal phenotype is incomplete or non‐specific. In the PAGE study results were analysed and returned after the end of the pregnancy but in clinical practice, where ES results will be returned rapidly in an ongoing pregnancy we need guidelines on reporting when the prenatal phenotype is incomplete and the phenotype–genotype correlation is uncertain. As experience with prenatal ES increases and the variations in prenatal phenotypes are further recognised, interpretation and reporting will become clearer.

### Disclosure of interests

RYE, DJM, MDK, ERM, MEH and LSC report grants from the Health Innovation Challenge Fund during the conduct of the PAGE study. DJM reports support from Congenica to attend educational symposia. MDK reports royalties or licenses from textbooks in fetal medicine, support from Illumina to attend a meeting on genomics in fetal medicine, and unpaid leadership roles on RCOG committees relating to genomics, all outside the submitted work. MEH reports patents, shares and personal fees from Congenica, outside the submitted work. LSC reports an unpaid leadership role as president of the ISPD (International Society for Prenatal Diagnosis). DBG reports consulting fees from AstraZeneca, Gossamer Bio, Gilead Sciences and GoldFinch Bio, and holds equity in Q State Biosciences, Praxis Therapeutics and Apostle Inc., all outside the submitted work. All other authors declare no competing interests. Completed disclosure of interests forms are available to view online as supporting information.

### Contribution to authorship

LSC contributed to the conception and design of this work, interpretation of data and drafting of the manuscript. RM contributed to study design, acquisition, analysis and interpretation of data and drafting the manuscript. JLG contributed to acquisition and interpretation of data and drafting the manuscript. RYE, SJH, DJM, MDK, ERM, MEH, VA, DBG and RJW contributed to acquisition and interpretation of data and revising of the manuscript. All authors approved the final version of this manuscript as submitted, and all authors agree to be accountable for all aspects of the work in ensuring that questions related to its accuracy or integrity are appropriately resolved.

### Details of ethics approval

The PAGE study received the ethical approval of research ethics committees at South Birmingham (approved 2 June 2014, REC reference number 14/WM/0150) and Harrow (approved 24 June 2014, REC reference number 01/0095) in the UK. The CUIMC study received the ethical approval of the institutional review board of Columbia University College of Physicians and Surgeons, New York, NY, USA (approved 23 February 2015, protocol #AAAO8009). For this analysis all clinical information was accessed in pseudo‐anonymised format, with participants’ written informed consent.

### Funding

The PAGE study was funded by the Health Innovation Challenge Fund (HICF) from the UK Department of Health and Wellcome Trust (no. HICF‐R7‐396). Additionally, RM is wholly funded and LSC is partially funded by the National Institute for Health Research (NIHR) Biomedical Research Centre at Great Ormond Street Hospital. LSC and ERM acknowledge support from the NIHR (Senior Investigator Award) and ERM acknowledges support from the NIHR Cambridge Biomedical Research Centre. The University of Cambridge has received salary support with regard to ERM from the UK NHS in the East of England through the Clinical Academic Reserve. The views expressed are those of the authors and not necessarily those of the NIHR, NHS or Department of Health. The CUIMC study was funded internally by the Department of OBGYN and the Institute for Genomic Medicine. Externalfunders of these studies had no role in study design, data collection, data analysis,data interpretation, or writing of the report.

### Acknowledgements

We thank the families who participated in the PAGE and CUIMC studies and the many research nurses and midwives and medical staff from UK fetal medicine units who contributed to recruitment and collection of clinical information for the PAGE study.

### Data Availability Statement

All genomic data from exome and genome sequencing (BAM files and VCF files) and clinical data files will be available through the European Genome‐phenome Archive (EGA) hosted by the European Bioinformatics Institute (EBI) at https://www.ebi.ac.uk/ega/.

## Supporting information

Supplementary Material

Supplementary Material

Supplementary Material

Supplementary Material

Supplementary Material

Supplementary Material

Supplementary Material

Supplementary Material

Supplementary Material

Supplementary Material

Supplementary Material

**Table S1**. Details of cases with non‐diagnostic but potentially clinically relevant variants, as judged by the PAGE study clinical review panel either due to variant of uncertain significance classification or uncertain contribution to phenotype.
**Table S2**. Postnatal outcomes for all fetuses initially presenting with increased NT at 11–14 weeks of gestation.
