# Supplementary material for: Fetal exome sequencing for isolated increased nuchal translucency: should we be doing it?
Source: BJOG. 2021 Sep 14;129(1):52–61. doi: 10.1111/1471-0528.16869 (PMC9292445; doi:10.1111/1471-0528.16869)
Supplement: Supplementary file 4 — Supplementary Material [file BJO-129-52-s010.pdf]

ICMJE DISCLOSURE FORM

Date: 19th May 2021

Your Name: Mark D. Kilby

Manuscript Title: Fetal exome sequencing for isolated increased nuchal translucency: Should we be doing it?
Manuscript number (if known): BJOG-21-0211

In the interest of transparency, we ask you to disclose all relationships/activities/interests listed below that are related to the content of your manuscript. "Related" means any relation with for-profit or not-for-profit third parties whose interests may be affected by the content of the manuscript. Disclosure represents a commitment to transparency and does not necessarily indicate a bias. If you are in doubt about whether to list a relationship/activity/interest, it preferable that you do so.

The following questions apply to the author's relationships/activities/interests as they relate to the current manuscript only.

The author's relationships/activities/interests should be defined broadly. For example, if your manuscript pertains to the epidemiology of hypertension, you should declare all relationships with manufacturers of antihypertensive medication, even that medication is not mentioned in the manuscript.

In item #1 below, report all support for the work reported in this manuscript without time limit. For all other items, the time frame for disclosure is the past 36 months.

		Name all entities with whom you have this relationship or indicate none (add rows as needed)	Specifications/Comments (e.g., if payments were made to you or to your institution)
		**Time frame: Since the initial planning of the work**	
1	All support for the present manuscript (e.g., funding, provision of study materials, medical writing, article processing charges, etc.) **No time limit for this item.**	None	X
		**Time frame: past 36 months**	
2	Grants or contracts from any entity (if not indicated in item #1 above).	None	X
3	Royalties or licenses	Cambridge University Press.	Fetal Therapy (2nd Edition)
		Taylor & Francis Group : CRC publishing	Obstetrics and Gynaecology : An evidenced based text for MRCOG (4th Edition)

4	Consulting fees	None	
5	Payment or honoraria for lectures, presentations, speakers bureaus, manuscript writing or educational events	____None	X
6	Payment for expert testimony	Perform Medicolegal work focused only on Fetal Medicine. 20 cases per annum	X
7	Support for attending meetings and/or travel	Illumina	Support to attend a meeting of fetal medicine subspecialists in EU to discuss Genomics in our specialty (Flights and accommodation). No payment or honoraria
8	Patents planned, issued or pending	____None	X
9	Participation on a Data Safety Monitoring Board or Advisory Board	____None	X
10	Leadership or fiduciary role in other board, society, committee or advocacy group, paid or unpaid	Chair of the Scientific Advisory Committee at RCOG	Unpaid
		Member of the RCOG Genomic Taskforce	Unpaid
		RCOG Representative on the Joint Colleges Committee on Genetics and Genomic Medicine	Unpaid
11	Stock or stock options	____None	X
12	Receipt of equipment, materials, drugs, medical writing, gifts or other services	____None	X
13	Other financial or non-financial interests	____None	X

Please place an "X" next to the following statement to indicate your agreement:

I certify that I have answered every question and have not altered the wording of any of the questions on this form.

Professor Mark Kilby 19th May 2021
