# Supplementary material for: Fetal exome sequencing for isolated increased nuchal translucency: should we be doing it?
Source: BJOG. 2021 Sep 14;129(1):52–61. doi: 10.1111/1471-0528.16869 (PMC9292445; doi:10.1111/1471-0528.16869)
Supplement: Supplementary file 12 — Table S1. Details of cases with non‐diagnostic but potentially clinically relevant variants, as judged by the PAGE study clinical review panel either due to variant of uncertain significance classification or uncertain contribution to phenotype. Table S2. Postnatal outcomes for all fetuses initially presenting with increased NT at 11–14 weeks of gestation. [file BJO-129-52-s003.docx]

**Table S1. Details of cases with non-diagnostic but potentially clinically relevant variants, as judged by the PAGE study clinical review panel either due to VUS classification or uncertain contribution to phenotype. † Variants previously published.**

DORV: double outlet right ventricle, Het: heterozygous, Hemi: hemizygous, Hom: homozygous, IUD: in utero demise, N/A: not applicable NAD: no abnormality detected, ToP: termination of pregnancy, VSD: ventricular septal defect, VUS: Variant of uncertain significance

| **Study Identifier** | **NT(mm)** | **Additional findings at presentation** | **Findings at later scans** | **Pregnancy outcome** | **Postnatal follow-up** | **Variant(s)**  **[Inheritance]** | **ACMG Classification** |
| --- | --- | --- | --- | --- | --- | --- | --- |
| PP0722 | 6.7 | None | NAD | Live birth | NAD at birth.  Follow up at 18m: fetal finger pads; sacral dimple; arched eyebrows | †*KMT2D* c.15535C>T p.(Arg5179Cys)  [Het, De novo] | Likely pathogenic |
| PP1723 | 5.0 | None | Nil structural (Ectopic heart beats only, not requiring treatment) | Live birth | Sacral dimple (no underlying neurological defect on MRI)  No hypertrichosis or dysmorphic features reported. | †*KMT2A* c.6526delG p.(Val2176Serfs*3)  [Het, De novo] | Likely pathogenic |
| PP1369 | 8.7 | None | Micrognathia (20/40) | IUD | Post-mortem: Dysmorphic facial features, prominent orbits, down-slanting palpebral fissures, low set ears, micrognathia, short neck, skin webs, petechiae of visceral pleura | †*NRAS* c.34G>A p.(Gly12Ser)  [Het, De novo] | Likely pathogenic |
| PP1528 | 9.3 | Hydrops (generalised oedema) | N/A (pregnancy ended) | ToP | Nil | †*PTPN11* c.1530G>T p.(Gln510His)  [Het, De novo] | VUS |
| PP3285 | 7.1 | Hypoplastic left heart | - | - | - | †*ACTB* c.193C>G p.(Leu65Val)  [Het, De novo] | Pathogenic |
| PP0312 | 4.8 | Hypoplastic left heart; DORV; transposition of great arteries; mega cisterna magna | N/A (pregnancy ended) | ToP | Nil | †*KIAA0586* c.1310A>T p.(Asp437Val)  [Hom, Biparental] | VUS |
| PP0206 | 11.3 | Hydrops; Cardiac ventricular disproportion R>L | Bilateral talipes; abnormal fixed flexion of extremities; reduced fetal activity; coarctation of aorta; possible VSD (16/40) | ToP | Post-mortem: Large cystic hygroma; widespread oedema; coarctation of aorta; small perimembranous VSD | †*SLC9A6* 375bp deletion  [Hemi, De novo] | Pathogenic |
| PP0585 | 4.6 | Generalised oedema | - | Live birth | - | †*ROBO1* c.1985delA  [Het, De novo] | VUS |

**Table S2. Postnatal outcomes for all fetuses initially presenting with increased NT at 11-14 weeks of gestation.**

ASD: atrial septal defect, DORV: double outlet right ventricle, Het: heterozygous, Hemi: hemizygous, Hom: homozygous, IUD: in utero demise, LTFU: lost to follow up, Mat: maternal, N/A: not applicable NAD: no abnormality detected, NND: neonatal death, Pat: paternal, PDA: patent ductus arteriosus, ToP: termination of pregnancy, VSD: ventricular septal defect, VUS: Variant of uncertain significance

| **Study Identifier** | **Diagnostic variant (s)**  **[Inheritance]** | **Pregnancy outcome** | **Postnatal follow-up** | **Final diagnosis** |
| --- | --- | --- | --- | --- |
| **Fetuses presenting with non-isolated increased NT at 11-14 weeks of gestation** | | | | |
| PP0342 | †*CHRNG* c.1010_1011del p.(His337Leufs*60) [Mat]  †*CHRNG* c.459dup p.(Val154Serfs*24) [Pat] | ToP | nil | Multiple pterygium syndrome |
| PP3174 | *TRIP11* c.757C>T p.(Arg253*) [Hom] | Miscarriage | nil | Achondrogenesis, type IA |
| PP1780 | †*TCTN2* c.1506-2A>G [Hom] | NND | nil | Joubert syndrome |
| PP2567 | †*PTPN11* c.922A>G p.(Asn308Asp) [Mat]  *SOS1* pathogenic variant [Pat] additionally detected on a postnatal RASopathy panel | NND | 33 weeks NND hydropic.  No post-mortem examination | Noonan syndrome |
| PP2000 | †*RYR1* c.7826C>A p.(Ser2609*) [Mat]  †*RYR1* c.10177_10198del p.(Leu3393CysfsTer25) [Pat] | Miscarriage | nil | Minicore myopathy with external ophthalmoplegia |
| PP4147 | *TCTN3* c.628-13_643del (splice variant) [Hom] | ToP | nil | Joubert syndrome |
| PP3324 | *BRAF* c.1782T>G p.(Asp594Glu) [De novo Het] | ToP | nil | Noonan syndrome |
| PP1843 | †*KMT2D* c.6295C>T p.(Arg2099*) [De novo Het] | ToP | Flat face, low set ears; loose neck skin, intestinal malrotation, horseshoe kidney; | Kabuki syndrome |
| PP3732 | *RYR1* 420bp deletion encompassing exon 29 [Hom] | ToP | No post-mortem examination | Minicore myopathy with external ophthalmoplegia |
| PP3393 | *GPC3* c.677delC p.(Thr226Ilefs*8) [Mat Hemi] | LTFU | nil | Simpson-Golabi-Behmel syndrome, type 1 |
| PP4393 | *RYR1* c.8342_8343delTA p.(Ile2781Argfs*49) [Pat]  *RYR1* c.2045G>A p.(Arg682Gln) [Mat] | ToP | Hydrops; flat nose, low-set ears; reduced muscle bulk; flexion deformities; | Minicore myopathy with external ophthalmoplegia |
| Fetal0183 | †*COL2A1* c.1358G>T p.(Gly453Val) [De novo Het] | ToP | nil | Type II collagenopathy |
| **Fetuses with initially isolated increased NT, then other anomalies detected later** | | | | |
| PP2904 | †*EPHB4* c.759dupC p.(Ser254Glnfs*10) [De novo Het] | IUD | No post-mortem examination | EPHB4-related lymphatic malformation |
| PP1726 | †*TAB2* c.1311_1312delTC p.(Pro438Glnfs*2) [De novo Het] | Live birth | Postnatal echocardiogram: mild tapering of aortic arch without coarctation; dysplastic tricuspid and pulmonary valves; bicuspid aortic valve.  At 4 and 12 months old: Hypotonia (mild); broad, upturned nose; prominent forehead; protruding tongue; webbing of toes; short stature. | TAB2-related congenital heart defect |
| PP0503 | †*PTPN11* c.922A>G p.(Asn308Asp) [Mat] | Live birth | - | Noonan syndrome |
| PP0692 | *RAF1* c.786T>G p.(Asn262Lys) [De novo Het] | Live birth | - | Noonan syndrome |
| PP1864 | †*KMT2D* c.673+1G>A [De novo Het] | ToP | Post-mortem: hypoplastic left heart with DORV, transposition of great arteries and pulmonary atresia | Kabuki syndrome |
| PP2033 | †*CHD7* c.656dupG p.(Leu220Profs*67) [De novo Het] | ToP | No post-mortem examination | CHARGE syndrome |
| PP1462 | †*BRAF* c.770A>G p.(Gln257Arg) [De novo Het] | NND | Premature delivery at 26/40. Subtle dysmorphic features; low-set ears; single palmar crease; severe pulmonary stenosis; PDA; ASD; short femurs.  Deceased due to sequelae of prematurity. No post-mortem examination. | Noonan syndrome |
| PP1807 | †*MID1* c.1102C>T p.(Arg368*) [De novo Hemi] | ToP | - | Opitz GBBB syndrome, type I |
| Fetal0116 | †*FLVCR2* c.1509+1G>A (splice variant) [Mat]  †*FLVCR2* c.1001dupT p.(Met334Ilefs*37) [Pat] | ToP | - | Proliferative vasculopathy and hydranencephaly-hydrocephaly syndrome (PVHH) |
| Fetal0222 | †*SOS1 c.1132A>G* p.(Thr378Ala) [Pat] | Live birth | - | Noonan syndrome |
| Fetal0307 | *NR2F2* c.1091delT p.(Leu364Cysfs*15) [De novo Het] | Live birth | - | NR2F2-related congenital heart defect |
| Fetal0385 | *FGD1* c.2026_2028delGAG p.(Glu676del) [Mat Hemi] | Live birth | - | Aarskog-Scott syndrome |
| **Fetuses with initially isolated increased NT which remained isolated or resolved later in pregnancy** | | | | |
| PP0602 | †Chr15 UPD [Mat] | Live birth | Low birth weight, no structural abnormalities detected at birth | Prader Willi syndrome |
| Fetal0045 | †*RERE c.248dupA* p.(Ser84Valfs*4) [De novo Het] | Live birth | No abnormalities detected at birth but at 8 months of age, length 25^th^ centile, weight 5^th^ centile, occipital frontal circumference (OFC) 25^th^ centile. Dysmorphic features including bifrontal narrowing, low anterior hairline, mild hypertelorism, bilateral epicanthal folds, downslanting palpebral fissures, synophrys, mild hypoplastic helices, redundant nuchal skin; spasticity of all four extremities with upper limbs more affected than lower limbs. | Neurodevelopmental disorder with or without anomalies of the brain, eye, or heart (NEDBEH) |
| **Fetuses presenting with initially isolated increased NT where later pregnancy follow up was not possible** | | | | |
| PP3321 | *PTPN11* c.214G>A p.(Ala72Thr) [De novo Het] | ToP | Post-mortem: cystic hygroma | Noonan syndrome |
| PP2039 | †*NIPBL* c.1435C>T p.(Arg479*) [De novo Het] | Miscarriage | Post-mortem: Small limbs. Characteristic facial gestalt appearance of Cornelia de Lange syndrome. No polydactyly. | Cornelia de Lange syndrome |
